# Relative performance of customized and universal probe sets in target enrichment: A case study in subtribe Malinae

**DOI:** 10.1002/aps3.11442

**Published:** 2021-07-23

**Authors:** Roman Ufimov, Vojtěch Zeisek, Soňa Píšová, William J. Baker, Tomáš Fér, Marcela van Loo, Christoph Dobeš, Roswitha Schmickl

**Affiliations:** ^1^ Department of Forest Growth, Silviculture and Genetics Austrian Research Centre for Forests Seckendorff‐Gudent‐Weg 8 Vienna 1130 Austria; ^2^ Komarov Botanical Institute Russian Academy of Sciences ul. Prof. Popova 2 St. Petersburg 197376 Russian Federation; ^3^ Institute of Botany The Czech Academy of Sciences Zámek 1 Průhonice 252 43 Czech Republic; ^4^ Department of Botany Faculty of Science Charles University Benátská 2 Prague 128 01 Czech Republic; ^5^ Royal Botanic Gardens, Kew Richmond Surrey TW9 3AE United Kingdom

**Keywords:** Angiosperms353, customized probe set, Malinae, target enrichment, universal probe set

## Abstract

**Premise:**

Custom probe design for target enrichment in phylogenetics is tedious and often hinders broader phylogenetic synthesis. The universal angiosperm probe set Angiosperms353 may be the solution. Here, we test the relative performance of Angiosperms353 on the Rosaceae subtribe Malinae in comparison with custom probes that we specifically designed for this clade. We then address the impact of bioinformatically altering the performance of Angiosperms353 by replacing the original probe sequences with orthologs extracted from the *Malus domestica* genome.

**Methods:**

To evaluate the relative performance of these probe sets, we compared the enrichment efficiency, locus recovery, alignment length, proportion of parsimony‐informative sites, proportion of potential paralogs, the topology and support of the resulting species trees, and the gene tree discordance.

**Results:**

Locus recovery was highest for our custom Malinae probe set, and replacing the original Angiosperms353 sequences with a *Malus* representative improved the locus recovery relative to Angiosperms353. The proportion of parsimony‐informative sites was similar between all probe sets, while the gene tree discordance was lower in the case of the custom probes.

**Discussion:**

A custom probe set benefits from data completeness and can be tailored toward the specificities of the project of choice; however, Angiosperms353 was equally as phylogenetically informative as the custom probes. We therefore recommend using both a custom probe set and Angiosperms353 to facilitate large‐scale systematic studies, where financially possible.

Phylogenetics has entered the era of target enrichment (Cronn et al., 2012), a method for isolating a specific set of loci from a DNA library using RNA or, less commonly, DNA baits. However, researchers must still choose which locus set to target in their study group. The use of universal probe sets for target enrichment, such as those for certain plant families (e.g., Mandel et al., 2014), the angiosperms (Angiosperms353; Johnson et al., 2019), or flagellate plants (Breinholt et al., 2021), standardizes the set of target loci and circumvents laborious probe design. Angiosperms353 is rapidly gaining popularity as a universal probe set for any angiosperm (e.g., rice [*Oryza sativa* L.] land races [Van Andel et al., 2019], *Nepenthes* L. [Murphy et al., 2020], *Cyperus* L. [Larridon et al., 2020], the Diapensiaceae [Gaynor et al., 2020], and *Schefflera* J. R. Forst. & G. Forst. [Shee et al., 2020]), with angiosperm probes for anchored hybrid enrichment being an alternative choice (Buddenhagen et al., 2016).

The Angiosperms353 markers seem to resolve the phylogenies of rapidly radiating groups well (Larridon et al., 2020; Shee et al., 2020) and show potential for resolving from deep (Johnson et al., 2019) to shallow phylogenetic scales, even resolving within‐species relationships (Van Andel et al., 2019; Murphy et al., 2020), but their utility has not yet been fully evaluated. To date, only one study has compared the benefits of Angiosperms353 with those of a custom probe set (Larridon et al., 2020). Two other studies that did not use Angiosperms353 as a universal probe set but compared the performance of universal probes with that of custom probes are Kadlec et al. (2017), who designed universal markers for *Erica* L. (Ericoideae), and Chau et al. (2018), who used three universal probe sets for *Buddleja* L. (Scrophulariaceae). All three studies, although utilizing different universal and custom probe sets, reported a similar degree of phylogenetic informativeness for both Angiosperms353 and the custom probe set. However, differences between these sets were also reported. Target locus recovery in outgroup taxa was more consistent for the universal probes than custom probes (Chau et al., 2018), due to a similar sequence divergence between probes and ingroup as well as outgroup taxa; for custom probes, the probe‐to‐outgroup sequence divergence was comparatively higher (e.g., Carlsen et al., 2018). On the other hand, the total number of target loci is usually higher in a custom probe set (Kadlec et al., 2017) because they are not constrained by the need to find conserved single‐copy loci across a large number of distantly related taxa. Locus recovery in the ingroup seemed to be generally higher for the custom probes, due to inherent features of the design of universal probes (Larridon et al., 2020). Universal and custom probe sets thus both have their advantages and disadvantages.

Increased data completeness through improved locus recovery could potentially be achieved by an optimization of the Angiosperms353 sequences toward a genome closely related to the study group, which would then be used as a reference for read mapping. To our knowledge, no one has yet attempted to optimize Angiosperms353 for target enrichment in the data analysis step, although the optimization of Angiosperms353 has recently been performed for the probe design (Jantzen et al., 2020).

In this study, we generated target enrichment data for the Rosaceae subtribe Malinae, with a particular focus on the genus *Crataegus* L., using both Angiosperms353 and our newly designed Malinae custom probe set (hereafter referred to as Malinae481) to evaluate the strengths and weaknesses of Angiosperms353. We were interested in the phylogenetic utility of Angiosperms353 from two angles: (i) in a comparison with Malinae481, and (ii) in a comparison with optimized Angiosperms353 sequences toward representatives from the *Malus domestica* (Suckow) Borkh. genome, which we used as a reference for read mapping.

## METHODS

### Taxonomic focus

The subtribe Malinae (tribe Maleae, family Rosaceae) includes up to 30 genera (Robertson et al., 1991) and over 10 hybrid genera, totaling more than 1000 species and interspecific as well as intergeneric hybrids (Phipps et al., 1990). Members of the Malinae natively occur mostly in the temperate zone of the Northern Hemisphere. According to both molecular (e.g., Potter et al., 2002) and morphological studies (e.g., Kalkman, 1988), the Malinae appear to be a monophyletic group. All members share the same base chromosome number (*x* = 17) and several characteristic traits, such as fruits with a varying degree of fleshiness derived from hypanthial ovaries, as well as widespread apomixis and polyploidy (Dickinson, 2018). It is hypothesized that a whole‐genome duplication (WGD) event followed by a rapid radiation played a central role in the origin of the group (Evans and Campbell, 2002; Velasco et al., 2010). The level of divergence between the genera of the Malinae is generally low, even though its origin dates back to at least the Middle Eocene (Lo and Donoghue, 2012); this explains the numerous intergeneric hybrids and the lack of resolution between its major clades.

Here, we selected 25 species within the Malinae, 13 in *Crataegus* and 12 belonging to seven genera from various other clades (Appendix 1). *Prunus tenella* Batsch from the tribe Amygdaleae was taken as an outgroup, although a rather divergent one. This sample set allowed us to test the relative performance of Angiosperms353 and Malinae481 at the subtribal and genus level.

### DNA ploidy estimation

We avoided neopolyploids in our sampling and therefore estimated the DNA ploidy level (Suda et al., 2006) of the various tissue samples (Appendix 1) using flow cytometry. The sample preparation followed the simplified two‐step protocol (Doležel et al., 2007). The seeds (rarely silica‐dried leaves) and an appropriate leaf volume of the internal standards (*Pisum sativum* L. cv. Ctirad [2C = 9.09 pg; Doležel et al., 1998] for Malinae; *Carex acutiformis* Ehrh. [2C = 0.82 pg; Lipnerová et al., 2013] for the outgroup *Prunus tenella*, which has a much smaller genome size) were chopped with a razor blade in a Petri dish containing 0.5 mL of ice‐cold Otto I buffer (0.1 M citric acid, 0.5% Tween 20). After a 10‐min incubation at room temperature, the suspension of nuclei was filtered through a 42‐μm nylon mesh. For the estimation of ploidy level, 1 mL of staining solution containing Otto II buffer (0.4 M Na_2_HPO_4_ · 12 H_2_O) and 4 μg/mL 4′,6‐diamidino‐2‐phenylindole (DAPI) was added to the suspension of nuclei. After a 5‐min incubation at room temperature, the solution of stained nuclei was analyzed using a CyFlow ML cytometer (Sysmex Partec, Görlitz, Germany) equipped with a 365‐nm UV‐LED as the source of UV light for DAPI excitation. The fluorescence intensity of 5000 particles was recorded for further data processing. We determined the ratio of DAPI‐stained nuclei in the G1 phase of the cell cycle (G1 peak) and the respective internal standards from the resulting histograms using the software FloMax FCS 2.0 (Sysmex Partec) to estimate the relative genome size. We then inferred the DNA ploidy level by comparing the relative genome sizes to the range of DNA amounts that Talent and Dickinson (2005) accepted as representing certain ploidy levels in *Crataegus*. These ploidy levels, together with genome sizes adopted from Talent and Dickinson (2005), are given in Appendix 1.

### Design of Malinae481

To design a custom probe set for the Malinae, we first evaluated the previously published divergence between single‐copy orthologs and relatively recently diverged paralogs in the genome of *M*. *domestica*. The average sequence divergence among orthologs was 3.65% in a genome‐wide comparison of *Malus* Mill. and *Pyrus* L. (Velasco et al., 2010), which is about one third of the average sequence divergence of 9.36% between the Maleae and Amygdaleae orthologs (Velasco et al., 2010). This divergence was approximately half as large as the 8% divergence between paralogs from the most recent WGD within each of these genomes (Wu et al., 2013), based on a four‐fold degenerate site transversion. Based on this information, we developed a strategy to identify in the *Malus* genome (1) single‐copy loci, presumably hidden paralogs due to the independent loss of distinct paralogs in different lineages, i.e., “one‐duplicate loss” (Xiang et al., 2017), and (2) loci only duplicated once (only one paralog present in the genome, avoiding multi‐gene families). We further constrained our locus selection by imposing a 6% minimum divergence between the orthologs and paralogs. Specifically, we BLASTed 28,695 mRNAs (referred to hereafter as queries) from the *M. domestica* ‘Golden Delicious’ genome version 1.1 (GDDH13; Daccord et al., 2017), downloaded from the Genome Database for Rosaceae (https://www.rosaceae.org [accessed November 2019]; Jung et al., 2019), against the *M. domestica* GDDH13 genome (referred to as the subject hereafter) using the nucleotide BLAST search. Default settings were used except for the *E*‐value, which was lowered to 0.00001. In an initial filtering step, we only retained the hits exceeding 70 bp and 10% of the query length with a ≥80% sequence similarity between the query and the subject. We then assigned the hits (usually corresponding to exons) to loci based on the criterion that the length of introns separating the hits did not exceed 10,000 bp. Queries that showed hits with more than six loci were not taken into account. In a second, refined filtering step, we retained only the loci that fulfilled the following criteria: length coverage and sequence similarity of the sum of all hits for a particular locus of ≥90%, length of single hits ≥100 bp (in accordance with the bait length of 100 bp), intron length ≤1200 bp, number of loci per query ≤2, and sequence divergence among loci ≥6%. We then BLASTed the 1280 *Malus* queries that fulfilled our selection criteria against the *Pyrus communis* L. Bartlett DH genome version 2.0 (Linsmith et al., 2019) and applied the same selection criteria, which 616 of these queries fulfilled.

Subject sequences from the *Malus* (799) and *Pyrus* (764) genomes, which matched the chosen mRNAs and represented full loci (including introns), were then extracted and the exon–intron boundaries were inferred based on the alignments, together with the BLASTed queries. After filtering for identical numbers of loci in both genomes, a sequence divergence between the exons of single‐copy *Malus* and *Pyrus* loci of ≤15%, and an exon length ≥80 bp, we ended up with 713 loci (481 loci if pairs of paralogous loci are treated as single loci), which corresponded to 546 mRNAs. All subject exons and introns were separated. Introns ≥80 bp in length were selected for bait design alongside the exons; however, the data set based on the targeted introns is not included in this study to ensure the comparability of the results based on Malinae481 with those of Angiosperms353, which are exclusively based on targeting exons. The extracted sequences were collapsed at ≥95% similarity and used for bait design. The final exonic probe set covers 2,008,479 bp in total.

### Illumina library preparation and target enrichment

The genomic library preparation followed two slightly different protocols for the different sequencing runs, which we indicate below. The affiliation of samples to sequencing runs is given in Appendix 1.

Genomic DNA was extracted using the DNeasy Plant Mini Kit (QIAGEN, Venlo, Netherlands). Between 200 ng and 1 µg of extracted DNA was sheared in 50 µL of double‐distilled water using an M220 Focused‐ultrasonicator (Covaris, Woburn, Massachusetts, USA) with the program for fragmentation to 500 bp for 25 s. Library preparation was performed using the NEBNext Ultra DNA Library Prep (New England Biolabs, Ipswich, Massachusetts, USA) protocol for Illumina with a few modifications: (1) a half volume of the samples and NEBNext chemicals were used during library preparation; (2) one additional cleanup step was implemented after the adapter ligation, for which a QIAquick PCR Purification Kit (QIAGEN) was used to clean residual nucleotides, enzymes, and salts from the DNA fragments as a prerequisite for efficient bead‐based size selection; (3) size selection (~400–600 bp) was performed using SPRIselect beads (Beckman Coulter, Brea, California, USA) with the ratio 0.65× for the left side and 0.55× for the right side selection, and amplification of the ligated size‐selected fragments was performed with eight cycles of PCR, using NEBNext Multiplex Oligos for Illumina Index Primers Set 1 and 2 (New England Biolabs) or Dual Index Set 1 (New England Biolabs); (4) enriched PCR products were cleaned twice with Agencourt AMPure XP beads (Beckman Coulter) with the ratios 0.75× and 0.7×. The libraries were subsequently pooled in approximately equimolar ratios in a 24‐plex (Angiosperms353) or 13‐plex (Malinae481) reaction.

We performed a solution hybridization using MyBaits biotinylated RNA baits (Arbor Biosciences, Ann Arbor, Michigan, USA). The enrichment followed the MYbaits manual version 3.02 (Angiosperms353) or version 4.01 (Malinae481) with approximately 700 ng (Angiosperms353) or 2 × 400 ng (Malinae481) input DNA and 12 cycles of PCR enrichment. Target‐enriched libraries were mixed with unenriched libraries (ratio 2 : 1) to increase the fraction of off‐target plastid reads, which tends to be small when using the most recent MYbaits kit versions. The majority of samples were sequenced on an Illumina (San Diego, California, USA) MiSeq at BIOCEV (Vestec, Czech Republic) using kit version 2 to obtain 250‐bp paired‐end reads. Together with 94 samples for a different study, two samples were sequenced on an Illumina NextSeq at the Genomics Core Facility of CEITEC (Brno, Czech Republic), utilizing the mid‐output kit to obtain 150‐bp paired‐end reads. All DNA concentration measurements were taken using a Qubit 2.0 fluorometer (Thermo Fisher Scientific, Waltham, Massachusetts, USA).

### Bioinformatic optimization of Angiosperms353 toward improved locus recovery

To improve the locus recovery from raw Angiosperms353 data, we created a reference for read mapping that was optimized for the Malinae. First, we needed to know the exon–intron boundaries of Angiosperms353, and thus BLASTed them against the genome of *M. domestica* GDDH13 using the National Center for Biotechnology Information (NCBI) Web BLAST with the ‘BLASTn’ default options (Johnson et al., 2008). The BLAST output was inspected using NCBI Genome Data Viewer (https://www.ncbi.nlm.nih.gov/genome/gdv/). Most of the probes did not have continuous hits, i.e., different parts of the same sequences usually had hits interrupted by intervals of various lengths. In addition, the exons sometimes appeared to be partial. Furthermore, we noticed a relatively low sequence similarity to the *Malus* genome. Around 80% of all sequence representatives of Angiosperms353 with hits showed a sequence similarity below 80%.

For the modification of the Angiosperms353 reference, two custom python scripts were employed (available from https://github.com/rufimov/2ex [accessed May 2020]). In the first step, using ‘2ex_extract.py’, we extracted all exons from the two *Malus* genomes (*M. domestica* ‘Golden Delicious’ [GDDH13] and ‘Hanfu’ [HFTH1; Zhang et al., 2019]), based on the genome annotation. These two genomes were selected because they differ in the quality and completeness of their assembly and annotation, which gave us a higher number of annotated genes when combining them. For the exon extraction, we included protein‐coding genes, long non‐coding RNAs, and pseudogenes (including transcribed ones), but we omitted all other types of RNAs, such as ribosomal RNAs, transfer RNAs, microRNAs, and small nucleolar RNAs, as well as plastid and mitochondrial sequences. The exons were then concatenated into transcripts, resulting in 67,246 transcripts for 43,659 loci in GDDH13 and 42,841 transcripts for 42,841 loci in HFTH1. Subsequently, using ‘2ex_split.py’, we BLASTed the Angiosperms353 sequences against the concatenated exons from GDDH13 and HFTH1, which were the outcome of the previous step, using standalone BLAST version 2.10.0+ (Camacho et al., 2009), and sorted the hit table according to hit length. By doing so, it was possible to obtain the top hit representative of each of the up to 18 representatives for each Angiosperms353 locus, as well as the best matching locus from either of the two *Malus* genomes. The best matching *Malus* loci were then split into individual exons based on the annotations, which were obtained in the beginning using ‘2ex_extract.py’, and they were BLASTed back against the best Angiosperms353 representatives using standalone BLAST. The resulting hit table was sorted according to hit length, and only the top hit for each exon was kept. The sequences of the best representatives of Angiosperms353 and the corresponding *Malus* exons were eventually trimmed to the size of the top hits (based on the start and end of hits) and subsequently concatenated.

Our bioinformatic optimization of Angiosperms353 resulted in two modified Angiosperms353 references with one sequence representative each per locus, and of relatively equal size (Table 1). One reference comprised the best single Angiosperms353 representative per locus (353 sequences with a total length of 225,060 bp; https://github.com/rufimov/2ex/blob/main/bestHit‐modified_Angiosperms353.fasta), whereas the other reference contained the original Angiosperms353 sequence representatives replaced by the best matching *Malus* sequence for each locus (353 sequences with a total length of 225,289 bp; https://github.com/rufimov/2ex/blob/main/Malinae‐optimized_Angiosperms353.fasta). We will refer to these references here as bestHit‐modified and Malinae‐optimized, respectively. The results based on the bestHit‐modified reference are presented, together with the results based on the other references (which we call probe sets if these sequences had initially been used for bait synthesis); this may help elucidate the reasons for any differences in the recovery of the target loci, if the number of sequence representatives per locus would play a role. If we had only compared the Malinae‐optimized reference with Angiosperms353, it could be argued that differences in locus recovery stem from the fact that the Angiosperms353 markers have multiple sequence representatives per locus, whereas the Malinae‐optimized reference has only one. All probe sets and references used in this study are summarized in Table 1.

**TABLE 1:** Overview of the probe sets for target enrichment and references for read mapping used in this study.

Characteristics of the probe set/reference	Malinae481	Angiosperms353	Malinae‐optimized	bestHit‐modified
Bait set	Malinae481	Angiosperms353	Angiosperms353	Angiosperms353
Applicability	Customized	Universal	Customized	Customized
No. of loci	481 if paralogous loci are treated as single loci; 713 if paralogous loci are represented as two loci	353	353	353
No. of sequence representatives per locus	2–4 if paralogous loci are treated as single loci; 2 if paralogous loci are represented as two loci	6–18 (mean 13.5)	1	1
Taxonomic affiliation of sequence representatives	*Malus domestica* GDDH13 and *Pyrus communis* Bartlett DH	Selected angiosperms	*Malus domestica* GDDH13 or HFTH1	One “best matching” out of the up to 18 angiosperm representatives

Note

GDDH13 = Golden Delicious; HFTH1 = Hanfu.

### Processing of target nuclear sequences

The raw data were pre‐processed using custom scripts (available at https://github.com/V‐Z/hybseq‐scripts [accessed May 2020]) and GNU parallel (Tange, 2018). The reads were trimmed using Trimmomatic version 0.39 (Bolger et al., 2014; SLIDINGWINDOW:5:20 LEADING:20 TRAILING:20 MINLEN:50). For deduplication, ‘clumpify.sh’ from BBmap version 38.42 (dedupe optical spany adjacent) was utilized. We then analyzed the pre‐processed reads using HybPiper version 1.3.1 (Johnson et al., 2016) with the Burrows–Wheeler aligner (BWA; Li and Durbin, 2009, 2010; Li, 2013) option using different reference files: up to four representatives per locus for Malinae481, up to 18 sequence representatives per locus for Angiosperms353, and a single sequence representative per locus for both Malinae‐optimized and bestHit‐modified. In the case of Malinae481, each pair of targeted paralogous loci was treated as a single locus. Read mapping was conducted using BWA and the contig assembly was performed using SPAdes (Bankevich et al., 2012). Subsequently, custom scripts were used to post‐process the HybPiper output, obtain assembly statistics, and align all contigs retrieved by HybPiper using MAFFT version 7.453 (Katoh and Standley, 2013) with the –auto and –adjustdirectionaccurately options. The packages ‘ape 5’ (Paradis and Schliep, 2019) and ‘ips’ (Heibl, 2008) in R version 3.6.2 (R Core Team, 2019) were used to trim all alignments (every row and then every column with more than 30% missing data were removed) and to obtain alignment statistics. Alignments containing fewer than four sequences were discarded. The alignment characteristics (number of taxa, alignment length, number and proportion of variable sites and parsimony‐informative [PI] sites) were calculated using AMAS version 0.98 (Borowiec, 2016). We finally utilized our scripts to estimate the gene trees in a maximum likelihood framework using IQTREE version 1.6.12 (Nguyen et al., 2015). ModelFinder (Kalyaanamoorthy et al., 2017) implemented in IQTREE was applied to determine the best‐fit model in combination with the invariable site plus FreeRate model to predict the sequence evolution for each gene, and 10,000 ultrafast bootstrap replicates (Hoang et al., 2018) were performed. The gene trees were rooted utilizing Newick Utilities version 1.6 (Junier and Zdobnov, 2010). The species tree was reconstructed using ASTRAL version 5.6.1 (Zhang et al., 2018) and rooted using Newick Utilities.

It should be noted that we did not remove potentially paralogous loci from our data sets. In a plant group such as the Malinae, the removal of paralogous loci resulting from the most recent WGD event would imply the omission of a large proportion of the data. Furthermore, if certain loci appear as single‐copy, it cannot be excluded that this is the result of one‐duplicate loss leading to hidden paralogy (Xiang et al., 2017). We overcame this dilemma by developing an approach of utilizing paralogs for phylogenetic reconstruction, which will be published in due course (Ufimov et al., in prep.); therefore, in this study, we only compared the proportion of potentially paralogous loci that were identified as such using HybPiper between all data sets.

We addressed topological conflict between gene trees and support for the species tree utilizing Phyparts (Smith et al., 2015). The resulting pie charts were mapped onto the species tree using ‘phypartspiecharts.py’ (available at https://github.com/mossmatters/MJPythonNotebooks [accessed May 2020]). Phyparts requires the rooting of the gene trees and species tree. As our trees were rooted using *Prunus tenella*, which was absent in certain gene trees, the number of gene trees was reduced to 350 for the Malinae481 data set and 330 for the Malinae‐optimized data set.

The completeness of target enrichment data sets may be influenced by the methods used for data analysis, particularly the read mapping approach (Larridon et al., 2020). We therefore compared HybPiper with the BWA option and the BLASTX (Altschul et al., 1990) option for read mapping (Table 2), in addition to comparing HybPiper, which uses read mapping and subsequent de novo assembly, with HybPhyloMaker (Fér and Schmickl, 2018), which is built on a reference‐guided read assembly. HybPiper showed a slightly better performance over HybPhyloMaker; all details about the HybPhyloMaker analyses can be found in Appendix 2.

**TABLE 2:** Assembly performance for 25 species within the Malinae and the outgroup *Prunus tenella* using the different probe sets/references and HybPiper, given for the exonic data set. All values are averaged across the species within the Malinae for each probe set/reference.

Probe set/reference	Malinae (25 species)								Outgroup (*Prunus tenella*)				
	Enrichment efficiency in mapped reads (%)	No. (%) of loci with zero data	No. (%) of loci with ≥25% target length^b^, ≥50% accessions presence^c^	No. (%) of loci with ≥25% target length, ≥75% accessions presence	No. (%) of loci with ≥50% target length, ≥50% accessions presence	No. (%) of loci with ≥50% target length, ≥75% accessions presence	No. (%) of loci with ≥75% target length, ≥50% accessions presence	No. (%) of loci with ≥75% target length, ≥75% accessions presence	Enrichment efficiency in mapped reads (%)	No. (%) of loci with zero data	No. (%) of loci with ≥25% target length	No. (%) of loci with ≥50% target length	No. (%) of loci with ≥75% target length
Malinae481	55.4%	0	478 (99.4%)	476 (99.0%)	477 (99.2%)	475 (98.8%)	475 (98.8%)	470 (97.7%)	43.4%	44 (9.1%)	305 (63.4%)	287 (59.7%)	247 (51.4%)
Angiosperms353 (BWA)^a^	25.0%	4 (1.1%)	329 (93.2%)	322 (91.2%)	304 (86.1%)	280 (79.3%)	218 (61.8%)	188 (53.3%)	22.7%	15 (4.2%)	336 (95.2%)	320 (90.7%)	257 (72.8%)
Angiosperms353 (BLASTX)^a^	11.5%	8 (2.3%)	331 (93.8%)	324 (91.8%)	314 (89.0%)	287 (81.3%)	248 (70.3%)	206 (58.4%)	14.9%	15 (4.3%)	336 (95.2%)	320 (90.7%)	257 (72.8%)
Malinae‐optimized	23.4%	2 (0.6%)	332 (94,1%)	324 (91.8%)	318 (90.1%)	305 (86.4%)	284 (80.5%)	257 (72.8%)	23.3%	9 (2.5%)	343 (97.2%)	334 (94.6%)	307 (87.0%)
bestHit‐modified	12.4%	74 (21.0%)	205 (58.0%)	185 (52.4%)	161 (45.6%)	132 (37.4%)	98 (27.8%)	81 (22.9%)	12.6%	116 (32.9%)	222 (62.9%)	188 (53.3%)	127 (36.0%)

Note

BWA = Burrows–Wheeler aligner.

^a^ In the case of Angiosperms353, we compared HybPiper with the BWA option and the BLASTX option for read mapping.

^b^ “Target length” refers to the recovered length per target locus. In the case of Angiosperms353, with multiple sequence representatives of differing lengths for each locus, the average length was calculated.

^c^ “Accessions presence” refers to the proportion of accessions with sequence information for each target locus.

### Processing off‐target plastid sequences

As plastid reads are a byproduct of target enrichment (Weitemier et al., 2014), we were interested in the recovery of the plastome using Angiosperms353 and Malinae481. BWA, implemented in HybPhyloMaker, was used to map the quality‐trimmed, deduplicated reads from all sequencing runs to the plastome of *M. angustifolia* (Aiton) Michx. (GenBank: NC_045410.1; Liu et al., 2019), from which we had removed one inverted repeat. For consensus calling using Kindel version 0.1.4 (Constantinides and Robertson, 2017), the minimum read depth was set to 2× and majority rule to 51%. To compare the number of plastid reads, we separately mapped the reads from the runs that used the same baits for target enrichment, but subsequently, because we used the same samples in the case of both bait sets, we merged all reads to infer the plastome phylogeny. This phylogeny was built from all coding regions and spacers/introns, which we extracted from the reference plastome based on its annotation. The resulting sequences were aligned using MAFFT version 7.029 and concatenated and partitioned using AMAS, so that each partition included either coding sequences or spacers/introns. In cases with multiple exons per gene, the exons were concatenated and partitioned as a whole. A model test was performed separately for all partitions using ModelTest‐NG version 0.1.6 (Darriba et al., 2020). The plastome tree was reconstructed using RAxML‐NG version 0.9.0 (Kozlov et al., 2019) with the best model for each partition and the bootstopping option with a maximum of 1000 bootstrap replicates. Bootstrapping converged after 150 replicates. The tree was rooted and visualized using FigTree version 1.4.4 (http://tree.bio.ed.ac.uk/software/figtree).

## RESULTS

### Ploidy estimation

A flow cytometric analysis resulted in high‐resolution histograms with the mean coefficient of variation (CV) of the G1 peak of the Malinae samples being 2.95% (range 2.20–3.45%) and 3.16% for *Prunus tenella*. The mean CV of G1 for the internal standard *Pisum sativum* was 1.66% (range 1.05–2.73%) and 1.50% for *Carex acutiformis*. Nevertheless, the ploidy level could be successfully determined from only 14 species within the Malinae (Appendix 1). These were found to be mainly diploid, except for a few *Crataegus* accessions, for which it was difficult to estimate the ploidy level due to an intermediate ratio between 2*x* and 3*x*. Moreover, the genome size and ploidy level of the four *Crataegus* samples were adopted from a previous study by Talent and Dickinson (2005); their genome sizes (ranging from 2C = 1.43 pg to 1.82 pg) suggested diploidy.

### Comparison of Angiosperms353 with Malinae481

**FIGURE 1:**
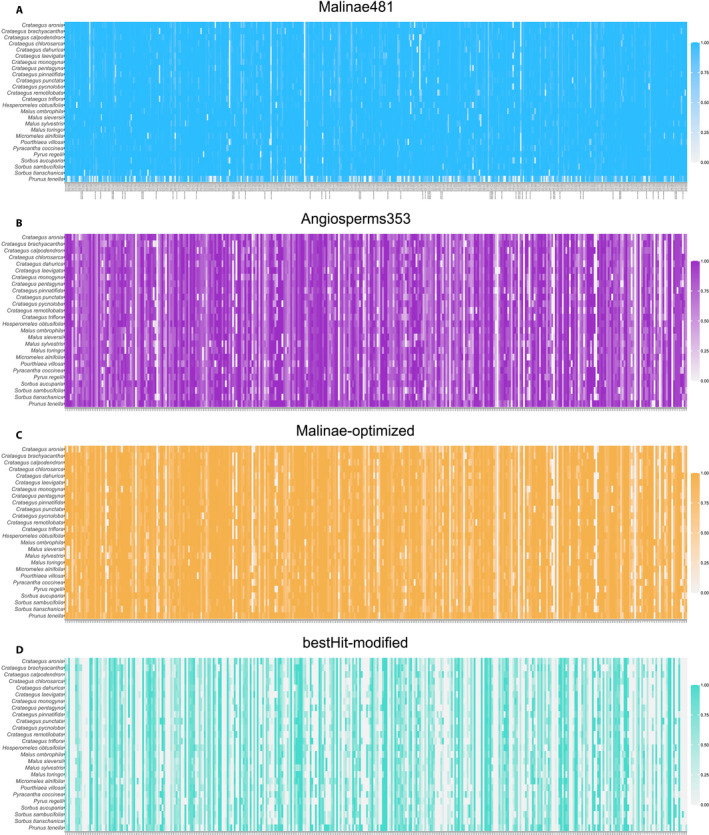
Heatmap of locus recovery using the different probe sets/references: Malinae481 (A), Angiosperms353 (B), Malinae‐optimized (C), bestHit‐modified (D). Each row represents a taxon, each column a locus. The color shading within each heatmap indicates the target length (i.e., recovered length per target locus).

Using HybPiper, the average assembly performance of Angiosperms353 and Malinae481 was compared (Table 2). Angiosperms353 resulted in 25.0% mapped reads for the Malinae and 22.7% mapped reads for the outgroup *Prunus tenella*, whereas Malinae481 allowed 55.4% and 43.4% of the reads to be mapped for the species within the Malinae and for the outgroup, respectively. Both probe sets showed a decrease in the number of loci when higher values of target length (i.e., recovered length per target locus) and accessions presence (i.e., proportion of accessions with sequence information per target locus) were applied, but with a different decline (Fig. 1, Table 2). This we found to be the most striking difference in the performance of Angiosperms353 vs. Malinae481. The best yield for species within the Malinae was obtained using Malinae481, for which the percentage of recovered loci was only slightly lower with a stricter missing data filtering, ranging from 99.4% to 97.7%, whereas Angiosperms353 showed a rapid decline for species within the Malinae (from 93.2% to 53.3% of recovered loci). For the outgroup, the percentage of recovered loci was much lower for Malinae481 (from 63.4% to 51.4%) than for Angiosperms353 (95.2% to 72.8%).

**FIGURE 2:**
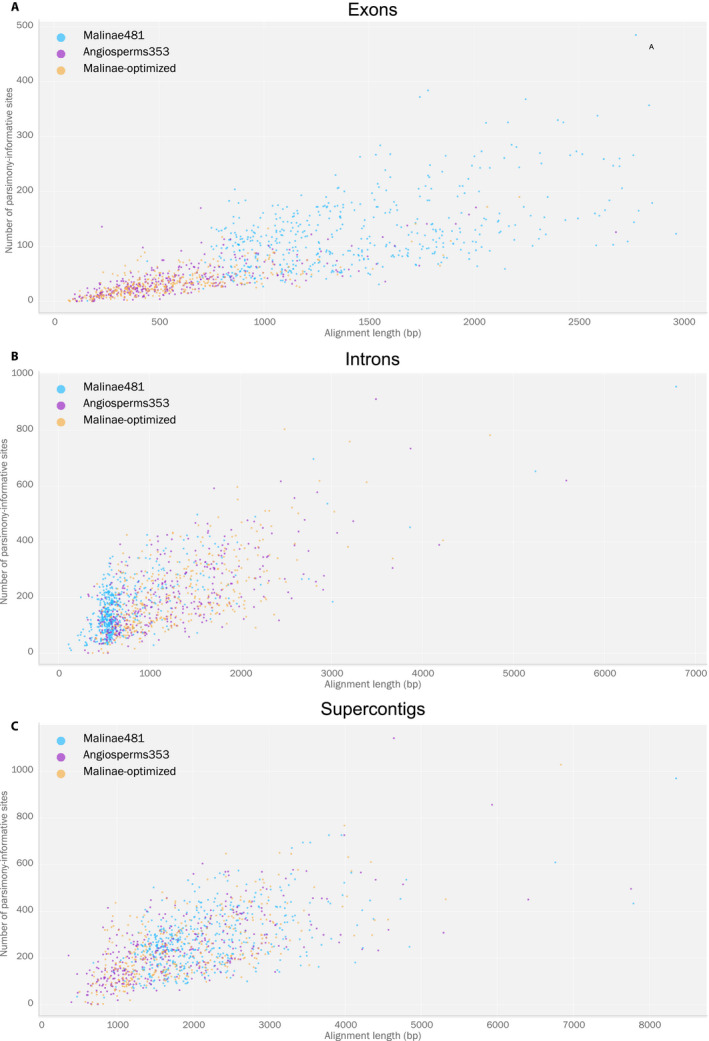
Scatter plot of alignment length vs. the number of parsimony‐informative sites for the exonic, intronic, and supercontig data sets using the different probe sets/references, excluding the bestHit‐modified reference. (A) Exons. (B) Introns. (C) Supercontigs.

The alignment characteristics for each data set (number of loci, average number of taxa per locus, alignment length, total alignment length, proportion of variable and PI sites per alignment, total number of PI sites) are provided for each probe set in Table 3, and the alignment length and proportion of PI sites are shown in Fig. 2. The number of loci and taxa were higher for Malinae481 (481 loci, 25 taxa; exonic data) than for Angiosperms353 (344 loci, 23 taxa; exonic data). The average and total alignment length were substantially lower when using Angiosperms353 compared with Malinae481 for the exonic data (average alignment lengths of 604 bp and 1415 bp, respectively; total alignment lengths of 207,717 bp and 680,658 bp, respectively), as was the supercontig data (average alignment lengths of 1850 bp and 2161 bp, respectively; total alignment lengths of 636,398 bp and 1,039,356 bp, respectively). In contrast, the intronic data set had higher values for Angiosperms353 than for Malinae481 (average alignment lengths of 1242 bp and 758 bp, respectively; total alignment lengths of 427,280 bp and 364,669 bp, respectively; Table 3). The proportion of variable and PI sites was similar between the Angiosperms353 and Malinae481, but differed between the exonic, intronic, and supercontig data sets (average proportion of PI sites: 6.7% and 9.0%, respectively, for exons; 18.0% and 23.8%, respectively, for introns; and 13.8% and 13.4%, respectively, for supercontigs; Table 3). The total number of PI sites was substantially higher for Malinae481 compared with Angiosperms353 for both the exonic (59,369 and 13,561, respectively) and supercontig data sets (133,632 and 82,339, respectively), although it must be acknowledged that the total locus number differed between Malinae481 (481 loci) and Angiosperms353 (353 loci).

**TABLE 3:** Alignment characteristics for 25 species within the Malinae and the outgroup *Prunus tenella* using the different probe sets/references and HybPiper, given for the exonic, intronic, and supercontig data sets. Alignment length, proportion of variable sites per alignment, and proportion of parsimony‐informative sites per alignment are given as minimum (min), average (avg), and maximum (max) values.

Data set	Probe set/ reference	No. of loci	Average no. of taxa per locus	Alignment length (bp) min/avg/max	Total alignment length (bp)	Proportion of variable sites per alignment [no. (%)] min/avg/max	Proportion of parsimony‐informative sites per alignment [no. (%)] min/avg/max	Total number of parsimony‐informative sites
Exons	Malinae481	481	25	159/ 1415/ 2964	680,658	96 (9.7%)/ 340 (24.4%)/ 945 (60.4%)	1 (0.6%)/ 123 (9.0%)/ 485 (23.7%)	59,369
	Angiosperms353	344	23	90/ 604/ 2677	207,717	1 (0.5%)/ 109 (18.1%)/ 586 (65.8%)	0/ 39 (6.7%)/ 171 (59.6%)	13,561
	Malinae‐optimized	346	24	69/ 575/ 2217	199,056	7 (2.3%)/ 103 (17.8%)/ 496 (45.0%)	0/ 35 (6.2%)/ 190 (22.6%)	12,151
	bestHit‐modified	256	20	69/ 421/ 1263	107,851	1 (0.8%)/ 69 (15.6%)/ 252 (56.0%)	0/ 25 (5.5%)/ 117 (18.2%)	6331
Introns	Malinae481	481	24	112/ 758/ 6789	364,669	50 (19.3%)/ 379 (53.2%)/ 1954 (88.3%)	11 (2.8%)/ 167 (23.8%)/ 957 (81.4%)	80,369
	Angiosperms353	344	22	292/ 1242/ 5582	427,280	27 (6.6%)/ 557 (46.6%)/ 2179 (85.7%)	1 (0.2%)/ 207 (18.0%)/ 912 (64.3%)	71,108
	Malinae‐optimized	347	24	376/ 1336/ 4743	463,577	18 (4.8%)/ 598 (46.1%)/ 1900 (78.2%)	1 (0.2%)/ 216 (16.9%)/ 804 (58.5%)	75,111
	bestHit‐modified	256	19	216/ 992/ 4041	254,030	19 (2.2%)/ 407 (41.5%)/ 1687 (84.6%)	1 (0.2%)/ 150 (15.6%)/ 597 (60.3%)	38,311
Supercontigs	Malinae481	481	25	475/ 2161/ 8353	1,039,356	201 (13.4%)/ 708 (33.6%)/ 2051 (56.6%)	31 (3.7%)/ 278 (13.4%)/ 970 (33.9%)	133,632
	Angiosperms353	344	23	363/ 1850/ 7759	636,398	35 (4.9%)/ 650 (36.3%)/ 2360 (72.9%)	2 (0.3%)/ 239 (13.8%)/ 1142 (58.4%)	82,339
	Malinae‐optimized	347	24	509/ 1902/ 6837	659,722	24 (2.9%)/ 678 (36.6%)/ 2376 (65.0%)	1 (0.2%)/ 245 (13.4%)/ 1029 (44.5%)	84,987
	bestHit‐modified	257	20	425/ 1401/ 5142	360,052	21 (2.2%)/ 468 (33.4%)/ 1863 (79.8%)	0/ 166 (11.9%)/ 642 (56.9%)	42,790

Approximately one third more loci were flagged by HybPiper as being potentially paralogous in the data set using Malinae481 (37.6%) compared with Angiosperms353 (24.2%; Appendix 3), which is the consequence of intentionally including paralogous loci when designing Malinae481.

### Bioinformatic optimization of the Angiosperms353 reference for the Malinae

To evaluate the outcome of our optimization of the original Angiosperms353 reference, the results based on the Malinae‐optimized reference were compared with those for Angiosperms353. The HybPiper results showed that the Malinae‐optimized reference performed similarly to Angiosperms353 (Fig. 1; Tables 2, 3), with the exception of the percentage of loci after missing data removal (Table 2): for the Malinae‐optimized reference, it slightly decreased with higher values of target length and accessions presence (from 94.1% to 72.8%), but it rapidly declined for Angiosperms353 (from 93.2% to 53.3%) when used for the Malinae. The decrease in data completeness was less pronounced for Angiosperms353 in the case of the outgroup (Fig. 1).

Alignment characteristics for concatenated exons, introns, and supercontigs are shown for Angiosperms353 and the Malinae‐optimized reference in Table 3 and Fig. 2. The number of loci and taxa were similar for Angiosperms353 and the Malinae‐optimized reference (344 loci and 23 taxa vs. 346 loci and 24 taxa, respectively; exonic data). The average alignment length was also comparable across all three data sets (604 bp and 575 bp, respectively, for exons; 1242 bp and 1336 bp, respectively, for introns; and 1850 bp and 1902 bp, respectively, for supercontigs). The proportion of variable and PI sites was slightly higher for Angiosperms353 than the Malinae‐optimized reference and differed between the exonic, intronic, and supercontig data sets (average proportion of PI sites: 6.7% and 6.2%, respectively, for exons; 18.0% and 16.9%, respectively, for introns; and 13.8% and 13.4%, respectively, for supercontigs; Table 3).

**FIGURE 3:**
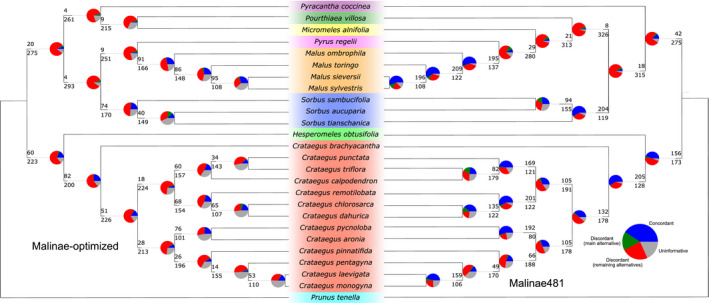
Comparison of topology and gene tree (in)congruence of ASTRAL species trees. The Malinae‐optimized reference and Malinae481 were used to generate these trees. For each branch, the top number indicates the number of gene trees concordant with the species tree at that node, and the bottom number indicates the number of gene trees in conflict with that node. The pie charts present the proportion of gene trees that support that clade (blue), the proportion that support the main alternative topology for that clade (green), the proportion that support the remaining alternative topologies (red), and the proportion that inform (support or conflict) that clade with <50% bootstrap support (gray).

The topologies of the species trees based on the Malinae‐optimized reference and Malinae481 differed slightly (Fig. 3, Appendix 4). The Malinae481 tree had substantially higher branch lengths and node support than the Malinae‐optimized tree (Appendix 4). The generally high gene tree discordance, which is common for target enrichment data sets (e.g., Morales‐Briones et al., 2018; Herrando‐Moraira et al., 2019), was lower for Malinae481 (Fig. 3).

As an intermediate step toward optimizing the Angiosperms353 reference for the Malinae, we chose the best hit of up to 18 sequence representatives of the Angiosperms353 loci for the *Malus* genome, which resulted in the bestHit‐modified reference (see the Methods section “Bioinformatic optimization of Angiosperms353 toward improved locus recovery”). We thereby evaluated the performance of this bestHit‐modified reference in comparison with Angiosperms353. The detailed assembly and alignment characteristics are presented in Tables 2 and 3. This reference performed by far the worst for all characteristics, which shows that the selected “best” Angiosperms353 sequence representative for each locus is in fact not the best because it still has a high sequence divergence from the reads; for example, only six representatives of the original Angiosperms353 are from the Malinae. Only in concert with other sequence representatives per locus can a satisfactory locus recovery be achieved.

More loci were flagged by HybPiper as potentially paralogous in the data set using the Malinae‐optimized reference (31.2%) compared with Angiosperms353 (24.2%; Appendix 3), which is probably a byproduct of the generally increased locus recovery in the case of the Malinae‐optimized reference (Table 3).

### Recovery of plastid data using Angiosperms353 vs. Malinae481

Plastome recovery was similar for the runs with Angiosperms353 and Malinae481 (Appendix 5); the average proportion of mapped plastid reads (1.9% and 2.6%, respectively) and the percentage of missing data (19.8% and 18.1%, respectively) showed little difference. When the reads from all runs were combined, the percentage of missing data dropped to a negligible 0.6%, and almost the entire plastome was successfully assembled for each accession.

The plastome tree showed high levels of support (Appendix 6) and had two main clades, similar to the multilocus nuclear tree: *Amelanchier*+*Crataegus* and *Malus*+*Pyrus*+*Sorbus*. The positions of *Pyracantha coccinea* M. Roem., *Micromeles alnifolia* Koehne, and *Pourthiaea villosa* Decne. were in discordance with the nuclear phylogeny; depending on the probe set used for target enrichment, the positions of these three taxa shift between clades. This may be due to the limited sampling in combination with the very short branches of the basal relationships.

## DISCUSSION

### Phylogenetic informativeness of Angiosperms353 vs. custom probes

Our results showed that universal probes and custom probes have a similar proportion of PI sites (Table 3), which is in agreement with the findings of Kadlec et al. (2017), Chau et al. (2018), Larridon et al. (2020), and Ogutcen et al. (2021). This seems to be a robust finding because both the universal and custom probe sets that were compared in the previous and present studies were generated in different ways and from different sources; only Larridon et al. (2020), Ogutcen et al. (2021), and this study used Angiosperms353 as the universal probe set. A similar proportion of PI sites in data sets generated with universal vs. custom probes was found across targeted plant groups from the genus level (*Erica* [Kadlec et al., 2017], *Buddleja* [Chau et al., 2018], *Cyperus* [Larridon et al., 2020], *Crataegus* [this study]) to the levels of subtribe (Malinae [this study]), family (Cyperaceae [Larridon et al., 2020], Gesneriaceae [Ogutcen et al., 2021]), and order (Ericales [Kadlec et al., 2017]). However, the scale to which phylogenetic informativeness applies may vary between universal and custom probe sets, with custom probes having a broader scale, from the infraspecific to the family level (Straub et al., 2020). Furthermore, phylogenetic informativeness per se does not guarantee the improvement of a phylogenetic hypothesis, as it is linked with data completeness, which we discuss below.

### Data completeness is high with custom probes

There are certain differences between universal and custom probes that deserve detailed consideration. The total number of loci in a custom probe set is often higher than in a universal probe set, e.g., 1164 (Schmickl et al., 2016) or 806 (Medina et al., 2019) (but see also smaller custom probe sets of 276 loci created by Nicholls et al. [2015] or of 176 loci in Heyduk et al. [2016]), which increases the total number of phylogenetically informative characters. This could be crucial for deciphering phylogenetic relationships between recently diverged taxa or within species, a future prospect of the target enrichment approach (Villaverde et al., 2018). However, even if the total locus number in a custom probe set is similar to a universal probe set, the average alignment length is often higher in custom probe sets, such as in the case of Malinae481: 604 bp is the average alignment length for Angiosperms353 in the Malinae, while for Malinae481 it is 1415 bp. Note that this length refers to the exonic data set, which resembles the sequences of Angiosperms353 and the majority of sequences of Malinae481 (we targeted introns as well, but did not include the data in this study). Interestingly, the average alignment length for both probe sets approximated each other for the supercontig data set due to increased intron recovery using Angiosperms353. This surprised us, as around 10% of Malinae481 were intronic sequences; thus, we expected a higher proportion of intronic data to be recovered, contributing to longer supercontigs. This suggests that explicitly targeting introns biased intron recovery toward the targeted introns, thereby limiting the total number of introns.

Another difference between universal and custom probes is the extent of missing data, which may differ between the in‐ and outgroups. Chau et al. (2018) emphasized that outgroup taxa have a similar data completeness to ingroup taxa when using universal probes, due to their more equal sequence similarity to both in‐ and outgroups in contrast with the custom probes. When comparing Angiosperms353 and Malinae481 in this respect, we did not find a pronounced difference. Regarding the ingroup, we observed a strong decrease in the number of recovered loci when applying the stricter missing data filter criteria for Angiosperms353 compared with Malinae481. This demonstrates that Angiosperms353, despite being as phylogenetically informative as custom probes, could be less informative for the ingroup because of a strongly reduced number of loci or a high degree of missing data if the phylogenetic reconstruction is performed on a highly fragmented data set. Nevertheless, Larridon et al. (2020) showed that the Angiosperms353 probe set has the potential to resolve rapid radiations, despite the above‐mentioned potential caveats. In addition, several phylogenomic studies support the notion that a larger fraction of missing data has no negative effect on phylogenetic reconstruction (Smith and Hahn, 2021, and references therein).

Data completeness may be influenced through the data analysis; for example, the read mapping approach seems to influence target enrichment efficiency (Jantzen et al., 2020; Larridon et al., 2020). BWA was superior to BLASTX in the case of custom probes, whereas for Angiosperms353, BLASTX was the better option as it permitted a lower sequence similarity for the matches, which is needed in the case of Angiosperms353 because the multiple sequence representatives for each locus are up to 30% diverged from each other (Johnson et al., 2019). Read mapping using BLASTX also improved the target enrichment efficiency in the case of the Malinae (Table 2); however, the optimization of Angiosperms353 gave a bigger improvement.

### Angiosperms353 optimization improved locus recovery

In the case of universal probe sets, another possibility for improving the recovery of target loci during data analysis could be an optimization of the probe sequences toward a genome closely related to the study group, which is then used as a reference for read mapping. Based on the genomic resources for the study group, the original sequences of a universal probe set could be replaced by appropriate orthologs from these sources, which we did for the Malinae. This should result in a higher sequence similarity between probes and reads, thereby leading to higher locus recovery. Although we did not detect a great improvement in the proportion of mapped reads when optimizing the Angiosperms353 reference using the *Malus* genome, we saw an increase in locus recovery: the percentage of loci with both a ≥75% target length and accessions presence was 53% for Angiosperms353 vs. 73% for the Malinae‐optimized reference. Bioinformatically altering the performance of Angiosperms353 could alternatively be achieved by increasing the number of taxa per locus in the reference file used for HybPiper analysis (McLay et al., 2021). Alternatively, the optimization of Angiosperms353 could be performed during probe design by replacing the Angiosperms353 sequences with appropriate orthologs from a genomic resource of the study group, such as genome skimming data (Jantzen et al., 2020), although the authors of the study emphasize that this genomic resource should be closely related to the study group.

### Unite them: Take the best from Angiosperms353 and a custom probe set

In summary, our results, taken together with insights from Larridon et al. (2020), clearly distinguish the pros and cons of Angiosperms353 and a custom probe set. The Angiosperms353 markers are powerful in elucidating the phylogenetic relationships among the angiosperms at various phylogenetic depths (Van Andel et al., 2019; Gaynor et al., 2020; Larridon et al., 2020; Murphy et al., 2020; Shee et al., 2020); thus, if a research group cannot afford to design a custom probe set, financially or bioinformatically, Angiosperms353 will likely be the best choice for angiosperm phylogenomics. Alternatively, if a research group can design custom probes, this will likely provide improved phylogenomic performance and allow them to address specific questions (e.g., by targeting specific genes or paralogs). We advise utilizing a combination of both probe sets, as shown by Hendriks et al. (2021) for a custom Brassicaceae probe set in combination with Angiosperms353, or, in case of financial restrictions, including appropriate orthologs for the Angiosperms353 loci when designing a custom probe set. Both of these approaches will enhance the access to outgroup data and create downstream opportunities for phylogenomic integration.

### A bright future for Malinae phylogenomics?

Our limited sample size does not allow for broad conclusions about possible improvements of the Malinae and *Crataegus* phylogeny using Angiosperms353, in particular their Malinae‐optimized version, as well as the use of Malinae481 for target enrichment. We built well‐resolved phylogenies, which are relatively consistent with the previously published Malinae phylogenies based on plastid and nuclear ITS data (Lo and Donoghue, 2012) and on plastome and nuclear ribosomal DNA cistron data (Liu et al., 2019, 2020), as well as the Malinae phylogeny produced as part of the Rosaceae phylogeny (Xiang et al., 2017). With the exception of the ambiguous placement of *Pyracantha* M. Roem., our plastome tree is identical to the ones obtained with a much broader taxon sampling. A comparison with the published nuclear phylogenies is less straightforward, as these are either not well resolved (Lo and Donoghue, 2012) or include an insufficient number of representatives (Xiang et al., 2017; Liu et al., 2019, 2020), but we see some similarities between them and the multilocus species trees presented in this study. The split between the *Crataegus*‐*Hesperomeles* clade and the majority of taxa within the Malinae is congruent with all previous studies, and the sister relationship of *Malus* and *Pyrus* coincides with the placement of these genera in the Rosaceae phylogeny of Xiang et al. (2017), which was based on low‐copy nuclear genes. Contrary to Lo and Donoghue (2012), but in congruence with the more recent studies cited above, *Pyracantha* is placed inside the Malinae. In general, the species tree topologies obtained using the Malinae‐optimized Angiosperms353 reference vs. Malinae481 are basically the same, with the exception of the position of *Pourthiaea* Decne. and *Micromeles* Decne. and the infrageneric relationships within *Crataegus* and *Sorbus* L. The lower gene tree discordance for Malinae481 in contrast with the Malinae‐optimized reference is probably the result of the slightly more informative Malinae481 loci, which are much longer on average. The phylogenetic relationships within *Crataegus* are generally in accordance with what we know so far about the evolution of this genus (Ufimov and Dickinson, 2020), giving partial support for the division of the genus into five subgenera, although our limited sampling prevents a detailed interpretation of the infrageneric relationships.

Our preliminary phylogenies must also be viewed with caution because of the insufficient processing of the paralogous loci that are the result of the most recent WGD in the evolution of the Malinae. The removal of the paralogous loci resulting from this WGD event would have depleted the majority of data, and we recognized the need to utilize paralogs for phylogenetic reconstruction; however, this is the focus of a follow‐up study (Ufimov et al., in prep.). In addition, the many auto‐ and allopolyploids in the Malinae require the proper discrimination between alleles and paralogs as the result of neopolyploidization or hybridization events. Such discrimination is particularly challenging when using short‐read Illumina data in combination with the usually short length of the targeted exons, but several recent phylogenomic studies using target enrichment have demonstrated that it is possible to overcome this challenge (Kamneva et al., 2017; Morales‐Briones et al., 2018), and the relatively long exons of our Malinae481 probes promise to be beneficial in this respect.

## AUTHOR CONTRIBUTIONS

**Roman Ufimov:** Formal analysis (equal); Methodology (equal); Software (equal); Visualization (equal); Writing – original draft (equal). **Vojtěch Zeisek:** Methodology (equal); Software (equal); Writing – review & editing (equal). **Soňa Píšová:** Formal analysis (equal); Investigation (equal); Writing – original draft (equal). **William J. Baker:** Resources (equal); Writing – review & editing (equal). **Tomáš Fér:** Methodology (equal); Writing – review & editing (equal). **Marcela van Loo:** Project administration (equal); Writing – review & editing (equal). **Christoph Dobeš:** Funding acquisition (equal); Methodology (equal); Project administration (equal); Supervision (equal). **Roswitha Schmickl:** Conceptualization (equal); Funding acquisition (equal); Project administration (equal); Supervision (equal); Writing – original draft (equal).

R.S. designed the study; C.D., R.S., and R.U. developed Malinae481; W.J.B. provided Angiosperms353; S.P. conducted the laboratory work; T.F. advised on the computational analyses; R.U., V.Z., and S.P. analyzed the data; and R.S., R.U., and S.P. wrote the initial draft of the manuscript. All authors contributed to and approved the final version of the manuscript.

## Data Availability

The exonic Malinae481 probe set is available at Dryad (https://doi.org/10.5061/dryad.j3tx95xc0; Dobeš et al., 2021). The reference comprising the best single Angiosperms353 representative per locus (https://github.com/rufimov/2ex/blob/main/bestHit‐modified_Angiosperms353.fasta) and the reference containing the original Angiosperms353 sequence representatives replaced by the best matching *Malus* sequence for each locus (https://github.com/rufimov/2ex/blob/main/Malinae‐optimized_Angiosperms353.fasta) are available at GitHub. Raw reads are available from the National Center for Biotechnology Information (NCBI) Sequence Read Archive (BioProject PRJNA668919).

## APPENDIX 1 Voucher, ploidy, and sequencing run information of the 25 Malinae accessions and the outgroup *Prunus tenella* used in this study.

**Table:**

Species	Ploidy level/ ratio to the internal standard or * genome size (2C value, pg)^a^	Type of material for target enrichment	Collection locality	Collection date	Collector/ collection number	Voucher	Sequencing platform, read length (bp) [Angiosperms353/ Malinae481]	NCBI SRA accession number (total number of reads) [Angiosperms353/ Malinae481]
*Crataegus aronia* (Willd.) Bosc		Herbarium	Greece, Crete, Iraklion province, Kato Asites, above St. George Gorgolaini monastery. Altitude 580–650 m a.s.l.	20 August 1987	K. I. Christensen, K. Bruhn Møller, A. Anagnostopoulos, and S. Diemar/ 634	LE 01021044	MiSeq, 250/ MiSeq, 250	SRR12879573 (1,274,080)/ SRR12958388 (1,333,160)
*Crataegus brachyacantha* Sarg. & Engelm.	2*x*–3*x*/ 1.73 ± 10*	Silica‐dried leaves	USA, Louisiana, Ouachita Parish, beside US165 S, 1.4 miles S of junction with I‐20 in Monroe, inside Richwood Corp. Limit. 32.476111°N, 92.083056°W	16 August 2004	C. Reid/ 5206	TRT 00000027	MiSeq, 250/ MiSeq, 250	SRR12879572 (1,037,126)/ SRR12958387 (1,423,198)
*Crataegus calpodendron* (Ehrh.) Medik.	2*x*–3*x*/ 1.82 ± 0.19*	Silica‐dried leaves	USA, Massachusetts, Suffolk Co., Boston, Jamaica Plain, Arnold Arboretum, Weld‐Walter Streets site. 42.303056°N, 71.124167°W	20 June 2002	T. A. Dickinson/ 2002‐07A	TRT 00000105	MiSeq, 250/ MiSeq, 250	SRR12879561 (1,238,568)/ SRR12958376 (1,299,918)
*Crataegus chlorosarca* Maxim.		Herbarium	Russian Federation, Petropavlovsk‐Kamchatsky, Rybakov prospekt, near building 19a	30 August 2018	D. Gimelbrant/ s.n.	LE 01020830	MiSeq, 250/ MiSeq, 250	SRR12879554 (1,092,632)/ SRR12958369 (980,132)
*Crataegus dahurica* Koehne ex C. K. Schneid.	2*x*/ 0.200	Herbarium	Russian Federation, Kemerovo Oblast, Kuzbass Botanical Garden, exposition ‘Ever‐blooming garden’. Provenance unknown	14 September 2016	V. Zagurskaya Iu. / 10	LE 01020939	MiSeq, 250/ MiSeq, 250	SRR12879553 (1,323,144)/ SRR12958368 (1,212,004)
*Crataegus laevigata* (Poir.) DC.	2*x*/ 0.187	Herbarium	Germany, Saarland, Schiffweiler, Heiligenwald, *Fraxinus excelsior* forest E Tafelbrunnen. Altitude 333 m a.s.l. 49.359834°N, 7.089546°E	24 April 2018	F.‐J. Weicherding/ 015/2018	WFBVA not barcoded	MiSeq, 250/ MiSeq, 250	SRR12879552 (1,176,534)/ SRR12958367 (1,155,916)
*Crataegus monogyna* Jacq.	2*x*/ 0.191	Herbarium	Germany, Saarland, Saarbrücken, Güdingen, shrubland along abandoned railway track. Altitude 194 m a.s.l. 49.205548°N, 7.020184°E	27 April 2018	F.‐J. Weicherding/ 014/2018	WFBVA not barcoded	MiSeq, 250/ MiSeq, 250	SRR12879551 (811,336)/ SRR12958366 (1,378,220)
*Crataegus pentagyna* Waldst. & Kit. ex Willd.	2*x*/ 0.194	Silica‐dried leaves	Republic of Crimea, Kirovskiy Rayon, vicinity of Stary Krym, tree thicket by Churuk‐su river. Altitude 290 m a.s.l. 45.02121°N, 35.09535°E	2 September 2016	A. Gnutikov and R. Ufimov/ 33.2	LE 01020926	MiSeq, 250/ MiSeq, 250	SRR12879550 (1,037,132)/ SRR12958365 (1,300,050)
*Crataegus pinnatifida* Bunge	2*x*/ 0.214	Silica‐dried leaves	Republic of Korea, Ganghwa‐do, Incheon, Ganghwa‐gun, Hwado‐myeon, Sagi‐ri, near Mani‐san, along roadside. Altitude 54 m a.s.l. 37.61113°N, 126.45354°E	21 September 2015	R. Ufimov/ s.n.	LE 01020531	NextSeq, 150/ MiSeq, 250	SRR12879549 (2,646,870)/ SRR12958364 (842,976)
*Crataegus punctata* Jacq.	2*x*/ 1.43 ± 0.12*	Silica‐dried leaves	Canada, Ontario, Bruce Co., Eastnor Twp., W Barrow Bay, E side Hwy 9 at S slope. Altitude 200 ft. 44.900000°N, 81.205556°W	7 September 1986	T. A. Dickinson/ D1378	TRT 00012528	MiSeq, 250/ MiSeq, 250	SRR12879548 (1,073,782)/ SRR12958363 (1,501,078)
*Crataegus pycnoloba* Boiss. & Heldr.		Herbarium	Greece, Arcadia province, Mt. Menalon, ski center above Kardaras. Altitude 1550–1700 m a.s.l.	28 August 1987	K. I. Christensen, K. Bruhn Møller, and A. Anagnostopoulos/ 1718	LE 01020857	MiSeq, 250/ MiSeq, 250	SRR12879571 (1,198,452)/ SRR12958386 (1,277,062)
*Crataegus remotilobata* Raikova ex Popov	2*x*–3*x*/ 0.255	Herbarium	Kazakhstan, Turkistan region, Sozak district, 7 km SW Taukent, Karatau Nature Reserve, NE slope of Mt. Bessaz, gorge of Itmuryn river. Altitude 1210 m a.s.l. 43.828153°N, 68.681692°E	10 June 2018	A. V. Grebenjuk/ 252ASM750	LE 01020824	MiSeq, 250/ MiSeq, 250	SRR12879570 (944,870)/ SRR12958385 (1,205,794)
*Crataegus triflora* Chapm.	2*x*–3*x*/ 1.73 ± 0.13*	Silica‐dried leaves	USA, Alabama, Autauga Co. Jones Bluff, SSW Peace, woods and prairie openings S of dirt road (Autauga Co. Rd. 9) running E from Autauga Co. Rd. 15, S of AL14 between Burnsville and Mulberry, S slope. Altitude 200 ft a.s.l. 32.398889°N, 86.779444°W	19 April 2003	N. Talent, S. Nguyen, T. A. Dickinson, and R. W. Lance/ 2003‐22	TRT 00021431	MiSeq, 250/ MiSeq, 250	SRR12879569 (1,284,680)/ SRR12958384 (1,696,294)
*Hesperomeles obtusifolia* (Pers.) Lindl.	2*x*/ 0.164	Silica‐dried leaves	Costa Rica, San José province, Páramo district, Pérez Zeledón canton, Cerro de la Muerte, Los Quetzales National Park, along main access road to ICE towers. Altitude 3389 m a.s.l. 9.565147°N, 83.755917°W	18 May 2016	T. A. Dickinson and A. K. Dickinson/ 2016‐03	LE 01020842	MiSeq, 250/ MiSeq, 250	SRR12879568 (1,305,802)/ SRR12958383 (1,172,096)
*Malus ombrophila* Hand.‐Mazz.	2*x*/ 0.176	Silica‐dried leaves	China, Yunnan, Lanping, Xue‐bang Shan, forest. Altitude 2500 m a.s.l.	9 August 2015	I. Illarionova, L. Wang, and T.‐J. Tong/ TM 1263	LE 01020832	MiSeq, 250/ MiSeq, 250	SRR12879567 (1,237,802)/ SRR12958382 (1,355,392)
*Malus sieversii* (Ledeb.) M. Roem.	2*x*/ 0.192	Herbarium	Kyrgyzstan, Chuy region, Jayyl (Kalinin) district, Tian Shan, N side of Kyrgyz Ala‐Too Range, Kara‐Balta river, on way out of Sosnovka gorge. Altitude 1180 m a.s.l. 42.639217°N, 73.896808°E	23 July 2018	A. V. Grebenjuk/ 385ASM1217–385ASM1233	LE not barcoded	MiSeq, 250/ MiSeq, 250	SRR12879565 (1,344,534)/ SRR12958380 (1,620,270)
*Malus sylvestris* (L.) Mill.	2*x*/ 0.180	Silica‐dried leaves	Russian Federation, Komarov Botanical Institute of the Russian Academy of Sciences, Arboretum, plot 126. Provenance: unknown.	2 October 2018	R. Ufimov/ 8	LE 01020853	MiSeq, 250/ MiSeq, 250	SRR12879564 (1,453,448)/ SRR12958379 (1,658,026)
*Malus toringo* (Siebold) Siebold ex de Vriese	2*x*/ 0.177	Silica‐dried leaves	Russian Federation, Komarov Botanical Institute of the Russian Academy of Sciences, Arboretum, plot 122. Provenance: Japan, Toyama Prefecture, Arimine lake. Altitude 1170 m a.s.l. 36.470833°N, 137.428889°E	2 October 2018	R. Ufimov/ 3	LE not barcoded	MiSeq, 250/ MiSeq, 250	SRR12879566 (1,198,030)/ SRR12958381 (991,498)
*Micromeles alnifolia* (Siebold & Zucc.) Koehne		Silica‐dried leaves	Republic of Korea, Jeju‐do, Jeju‐si, Aewol‐eup, Eoeum‐ri. Altitude 610 m a.s.l. 33.37611°N, 126.39333°E	15 October 2018	R. Ufimov and I. Tatanov/ 12‐3	LE not barcoded	MiSeq, 250/ MiSeq, 250	SRR12879563 (1,221,564)/ SRR12958378 (1,214,624)
*Pourthiaea villosa* (Thunb.) Decne.		Silica‐dried leaves	Republic of Korea, Jeju‐do, Jeju‐si, Aewol‐eup, Eoeum‐ri. Altitude 705 m a.s.l. 33.37861°N, 126.38917°E	15 October 2018	R. Ufimov and I. Tatanov/ 14‐15	LE not barcoded	MiSeq, 250/ MiSeq, 250	SRR12879562 (1,132,796)/ SRR12958377 (1,173,788)
*Pyracantha coccinea* M. Roem.	2*x*/ 0.173	Silica‐dried leaves	Russian Federation, Stavropol Krai, Pyatigorsk, research station of Komarov Botanical Institute of the Russian Academy of Sciences, Perkalskiy Arboretum. Provenance: unknown	27 October 2018	Z. Dutova/ s.n.	LE 01020851	MiSeq, 250/ MiSeq, 250	SRR12879559 (1,261,066)/ SRR12958374 (1,556,426)
*Pyrus regelii* Rehder	2*x*/ 0.147	Herbarium	Kazakhstan, Turkistan region, Akimat of Kentau, 10 km NNE Kentau, Karatau Nature Reserve, gorge of Byresik river, 1–1.5 km upstream river mouth. Altitude 775 m a.s.l. 43.601344°N, 68.602367°E	27 May 2018	A. V. Grebenjuk/ 192KAZ524‐527	LE not barcoded	MiSeq, 250/ MiSeq, 250	SRR12879558 (1,146,976)/ SRR12958373 (1,585,440)
*Sorbus aucuparia* L.		Silica‐dried leaves	Russian Federation, Saint Petersburg, Krasnoselsky Rayon, Duderhof heights, Orekhovaya Gora, Nagorny park. 59.698476°N, 30.127742°E	29 September 2018	R. Ufimov/ 2	LE 01020844	MiSeq, 250/ MiSeq, 250	SRR12879557 (1,195,478)/ SRR12958372 (1,016,402)
*Sorbus sambucifolia* (Cham. & Schltdl.) M. Roem.		Silica‐dried buds	Russian Federation, Komarov Botanical Institute of the Russian Academy of Sciences, Arboretum, plot 131. Provenance: Sakhalin Oblast, wild origin	6 December 2018	R. Ufimov/ s.n	Unvouchered	MiSeq, 250/ MiSeq, 250	SRR12879556 (1,446,204)/ SRR12958371 (1,148,290)
*Sorbus tianschanica* Rupr.	2*x*/ 0.192	Herbarium	Kyrgyzstan, Chuy region, Jayyl (Kalinin) district, Suusamyr Aiyl Okmotu, Tian Shan, W edge of Jumgal‐Too Range, Kökömeren river, near confluence of Suusamyr and Zapadniy Karakol rivers. Altitude 1995 m a.s.l. 42.093169°N, 74.123264°E	31 July 2018	A. V. Grebenjuk/ 385ASM1217–385ASM1233	LE not barcoded	MiSeq, 250/ MiSeq, 250	SRR12879555 (1,179,900)/ SRR12958370 (1,034,408)
*Prunus tenella* Batsch	2*x*/ 0.446	Fresh buds	Czech Republic, Prague, Charles University, Botanical Garden of the Faculty of Science, Central European Flora section (calcareous vegetation), ACCID: 2007.02068. Provenance: unknown	8 February 2019	S. Píšová/ s.n.	Unvouchered	NextSeq, 150/ MiSeq, 250	SRR12879560 (3,510,486)/ SRR12958375 (1,205,220)

Note

NCBI SRA = National Center for Biotechnology Information Sequence Read Archive.

^a^ The genome size (2C value, pg) of samples from Talent and Dickinson (2005) are marked with an asterisk. Ploidy was estimated mainly from seed isolates. If seeds were not available, ploidy estimates were obtained from silica‐dried leaves; however, these were of insufficient quality for ploidy estimation in a few cases.

## APPENDIX 2 Assembly performance for 25 species within the Malinae and the outgroup *Prunus tenella* using the different probe sets/references and HybPhyloMaker, given for the exonic data set. All values are averaged across the species within the Malinae for each probe set/reference.

**Table:**

Probe set/reference	Malinae (25 species)								Outgroup (*Prunus tenella*)				
	Enrichment efficiency in mapped reads^a^ (%)	No. (%) of loci with zero data	No. (%) of loci with ≥25% missing data^b^, ≥50% species presence^c^	No. (%) of loci with ≥25% missing data, ≥75% species presence	No. (%) of loci with ≥50% missing data, ≥50% species presence	No. (%) of loci with ≥50% missing data, ≥75% species presence	No. (%) of loci with ≥75% missing data, ≥50% species presence	No. (%) of loci with ≥75% missing data, ≥75% species presence	Enrichment efficiency in mapped reads (%)	No. (%) of loci with zero data	No. (%) of loci with ≥25% missing data	No. (%) of loci with ≥50% missing data	No. (%) of loci with ≥75% missing data
Malinae481	52.6%	0	479 (99.6%)	478 (99.4%)	478 (99.4%)	469 (97.5%)	469 (97.5%)	463 (96.3%)	39.9%	29 (6.0%)	418 (86.9%)	350 (72.8%)	214 (44.5%)
Malinae‐optimized	22.2%	2 (0.5%)	338 (95.8%)	336 (95.2%)	322 (91.2%)	313 (88.7%)	270 (76.5%)	239 (67.7%)	22.4%	9 (2.5%)	338 (95.8%)	322 (91.2%)	218 (61.8%)
bestHit‐modified	11.3%	62 (17.6%)	148 (41.9%)	132 (37.4%)	66 (18.7%)	58 (16.4%)	32 (9.1%)	27 (7.6%)	11.9%	92 (26.1%)	161 (45.6%)	69 (19.5%)	28 (7.9%)

^a^ Raw reads were processed using HybPhyloMaker version 1.6.4 (Fér and Schmickl, 2018). The following parameter options were taken: Reads were quality‐trimmed using Trimmomatic version 0.32 (LEADING:20 TRAILING:20 SLIDINGWINDOW:5:20 MINLEN:36). Duplicate reads were removed utilizing FastUniq version 1.1 (Xu et al., 2012). Subsequently, the reads were mapped to a “pseudoreference,” comprising the exonic probe sequences divided by a stretch of 800 Ns between each exon, using BWA. Three pseudoreferences were built from Malinae481, in addition to the Malinae‐optimized and bestHit‐modified reference sequences. As HybPhyloMaker allows only one sequence representative per locus, we used one of the two copies in the case of the paralogous loci of Malinae481, from the *Malus* sequence representatives. The 51% majority consensus sequences were generated with a minimum read depth of 8× using Kindel version 0.1.4 (Constantinides and Robertson, 2017). Consensus sequences were matched to the probe sequences using BLAT (Kent, 2002) with 80% minimum sequence similarity. To compare the assembly performance between the different reference sequences for read mapping and between HybPhyloMaker and HybPiper, six combinations of filter criteria were applied: ≤75%, ≤50%, and ≤25% of missing data per accession in each alignment, and ≥50% and ≥75% of accessions with sequence information for each alignment.

^b^ “Missing data” refers to the proportion of missing data per accession in each alignment.

^c^ “Species presence” refers to the proportion of accessions with sequence information per each alignment.

## APPENDIX 3 Number and percentage of loci flagged as potentially paralogous by HybPiper for 25 species within the Malinae and the outgroup *Prunus tenella* using the different probe sets/references.

**Table:**

	No. (%) of loci			
Species	Malinae481	Angiosperms353	Malinae‐optimized	bestHit‐modified
*Crataegus aronia*	168 (47.6%)	72 (20.4%)	92 (26.1%)	34 (9.6%)
*Crataegus brachyacantha*	174 (49.3%)	86 (24.4%)	110 (31.2%)	32 (9.1%)
*Crataegus calpodendron*	171 (48.4%)	82 (23.2%)	103 (29.2%)	32 (9.1%)
*Crataegus chlorosarca*	168 (47.6%)	81 (23.0%)	96 (27.2%)	28 (7.9%)
*Crataegus dahurica*	172 (48.7%)	72 (20.4%)	97 (27.5%)	29 (8.2%)
*Crataegus laevigata*	167 (47.3%)	81 (23.0%)	105 (29.8%)	31 (8.8%)
*Crataegus monogyna*	179 (50.7%)	82 (23.2%)	97 (27.5%)	34 (9.6%)
*Crataegus pentagyna*	177 (50.1%)	84 (23.8%)	109 (30.9%)	31 (8.8%)
*Crataegus pinnatifida*	164 (46.5%)	53 (15.0%)	81 (23.0%)	25 (7.1%)
*Crataegus punctata*	176 (49.9%)	88 (24.9%)	106 (30.0%)	35 (9.9%)
*Crataegus pycnoloba*	172 (48.7%)	83 (23.5%)	105 (29.8%)	38 (10.8%)
*Crataegus remotilobata*	172 (48.7%)	80 (22.7%)	93 (26.4%)	35 (9.9%)
*Crataegus triflora*	174 (49.3%)	81 (23.0%)	104 (29.5%)	33 (9.4%)
*Hesperomeles obtusifolia*	182 (51.6%)	98 (27.8%)	132 (37.4%)	45 (12.8%)
*Malus ombrophila*	198 (56.1%)	98 (27.8%)	128 (36.3%)	42 (11.9%)
*Malus sieversii*	192 (54.4%)	97 (27.5%)	121 (34.3%)	44 (12.5%)
*Malus sylvestris*	185 (52.4%)	86 (24.4%)	121 (34.3%)	41 (11.6%)
*Malus toringo*	188 (53.3%)	81 (23.0%)	117 (33.1%)	35 (9.9%)
*Micromeles alnifolia*	183 (51.8%)	94 (26.6%)	126 (35.7%)	34 (9.6%)
*Pourthiaea villosa*	190 (53.8%)	83 (23.5%)	109 (30.9%)	36 (10.2%)
*Pyracantha coccinea*	195 (55.2%)	85 (24.1%)	112 (31.7%)	38 (10.8%)
*Pyrus regelii*	196 (55.5%)	92 (26.1%)	119 (33.7%)	38 (10.8%)
*Sorbus aucuparia*	191 (54.1%)	109 (30.9%)	139 (39.4%)	44 (12.5%)
*Sorbus sambucifolia*	199 (56.4%)	96 (27.2%)	125 (35.4%)	42 (11.9%)
*Sorbus tianschanica*	196 (55.5%)	94 (26.6%)	120 (34.0%)	35 (9.9%)
**Mean Malinae**	**181.2 (37.6%)**	**85.5 (24.2%)**	**110.7 (31.2%)**	**35.6 (10.1%)**
*Prunus tenella*	11 (3.1%)	5 (1.4%)	2 (0.6%)	0

## APPENDIX 5 Off‐target plastid read recovery, using the two different probe sets for target enrichment (Angiosperms353 and Malinae481).

**Table:**

Probe sets	Average mapped reads (%)	Missing data (%)
Angiosperms353	1.9	19.8
Malinae481	2.6	18.1
Combined	2.3	0.6
